# Complement Component C5a and Fungal Pathogen Induce Diverse Responses through Crosstalk between Transient Receptor Potential Channel (TRPs) Subtypes in Human Conjunctival Epithelial Cells

**DOI:** 10.3390/cells13161329

**Published:** 2024-08-09

**Authors:** Loreena Rech, Tina Dietrich-Ntoukas, Peter S. Reinach, Tobias Brockmann, Uwe Pleyer, Stefan Mergler

**Affiliations:** 1 Department of Ophthalmology, Charité—Universitätsmedizin Berlin, Corporate Member of Freie Universität Berlin and Humboldt-Universität zu Berlin, 10117 Berlin, Germany; loreena.rech@charite.de (L.R.); tina.dietrich-ntoukas@charite.de (T.D.-N.); uwe.pleyer@charite.de (U.P.); 2 School of Ophthalmology and Optometry, Wenzhou Medical University, Wenzhou 325015, China; preinach25@gmail.com; 3 Department of Ophthalmology, Universitätsmedizin Rostock, 18057 Rostock, Germany; tobias.brockmann@med.uni-rostock.de; 4 SciTec Department, University of Applied Sciences Jena, 07745 Jena, Germany

**Keywords:** complement factor C5a, fungal pathogen, transient receptor potential channel vanilloid 1, transient receptor potential channel melastatin 8, conjunctival epithelial cells, Ca^2+^ signaling

## Abstract

The conjunctiva has immune-responsive properties to protect the eye from infections. Its innate immune system reacts against external pathogens, such as fungi. The complement factor C5a is an important contributor to the initial immune response. It is known that activation of transient-receptor-potential-vanilloid 1 (TRPV1) and TRP-melastatin 8 (TRPM8) channels is involved in different immune reactions and inflammation in the human body. The aim of this study was to determine if C5a and *mucor racemosus e voluminae cellulae* (MR) modulate Ca^2+^-signaling through changes in TRPs activity in human conjunctival epithelial cells (HCjECs). Furthermore, crosstalk was examined between C5a and MR in mediating calcium regulation. Intracellular Ca^2+^-concentration ([Ca^2+^]_i_) was measured by fluorescence calcium imaging, and whole-cell currents were recorded using the planar-patch-clamp technique. MR was used as a purified extract. Application of C5a (0.05–50 ng/mL) increased both [Ca^2+^]_i_ and whole-cell currents, which were suppressed by either the TRPV1-blocker AMG 9810 or the TRPM8-blocker AMTB (both 20 µM). The N-terminal peptide C5L2p (20–50 ng/mL) blocked rises in [Ca^2+^]_i_ induced by C5a. Moreover, the MR-induced rise in Ca^2+^-influx was suppressed by AMG 9810 and AMTB, as well as 0.05 ng/mL C5a. In conclusion, crosstalk between C5a and MR controls human conjunctival cell function through modulating interactions between TRPV1 and TRPM8 channel activity.

## 1. Introduction

Conjunctiva is an immune-responsive tissue, covering the ocular surface and the inner parts of the lower and upper eyelids. This human mucosa is constantly exposed to an environment that is laden with potentially infective and allergenic substances. The epithelial layer of the cornea and the conjunctiva function as a natural barrier against pathogenic infiltration. The tear film, which covers the corneal and conjunctival epithelium, has many properties that act against external pathogens. The corneal stroma is in healthy condition, free of lymphatic vessels and tissues. It only contains a low number of immunoreactive cells, whereas the conjunctiva has conjunctival-associated lymphatic tissue (CALT) [1,2]. The conjunctiva can be compromised by inflammation and allergic reactions caused by pathogens and allergens in the environment. Inflammation of the conjunctiva can be classified as non-infectious, e.g., allergies, or infectious, for example, by viruses, bacteria, or fungi. Common symptoms are red, itchy, and painful eyes that are watery or even purulent [3].

The fungus *Mucor racemosus* is commonly found in dirt and rotten foods [4]. In this study, *Mucor racemosus e voluminae cellulae* (MR) was used, which is an active ingredient obtained from the mold *Mucor racemosus*. The spore-forming zygomycota from the order mucorales can live in versatile conditions, either in the form of yeast or mold, with the ability to survive at temperatures higher than 37 °C. It can cause severe human infections such as rhino-orbital-cerebral mucormycosis and allergic reactions like rhino-conjunctivitis [5,6,7,8]. Rhino-orbital-cerebral mucormycosis is an infection caused by spores of mold, often starting in the form of sinusitis and migrating to the eye socket and brain [5,9,10]. Infection can end in blindness or even death and is related to risk factors such as immunosuppression and diabetes [9,11].

The complement system is an integral part of the innate humoral immune system. It initiates inflammation by activating the complement cascade. The result is the formation of a membrane attack complex (MAC) to destroy pathogens [7,12]. The anaphylatoxin C5a, a fragment from proteolytic scission of complement factor C5, has a chemotactic effect via diapedeses on other immune cells such as monocytes, granulocytes, or mast cells. Furthermore, the proinflammatory glycoprotein promotes the production and release of chemokines as well as cytokines (IL-1β, IL-6, TNF, and MIP) from recruited immune cells and assists oxidative burst [13,14,15,16]. Three G protein-coupled receptors (GPCRs) for C5a are known: C5a receptor 1 (C5aR1) (C5a anaphylatoxin chemotactic receptor 1/CD88), as well as the not yet well described C5L2 (C5aR2), but also C3aR [17]. C5aR1 regulates C5a-induced proinflammatory processes. In contrast, C5aR2 (C5L2) was for a long time classified as a competitive decoy-receptor inhibiting proinflammatory reactions [18]. Notably, this response to C5a is mediated through C5aR1 activation of an unknown intracellular Ca^2+^-influx pathway [15]. On the other hand, TRPV1 activation mediates hypersensitivity responses to C5a activation [19,20]. C5a is known to be active in the human eye, especially during the night [21]. Regarding other ocular conditions, it is still unclear if C5a activation underlies the pathogenesis, such as in age-related macular degeneration [22,23], sicca-syndrome [24] or mucous membrane pemphigoid [25], yet it is highly probable.

Calcium is an important first and second messenger for mediating receptor control of many cellular processes [26,27,28]. Those processes are dependent on the calcium gradient, which is controlled by channels in the membranes of cells and organelles [29]. Transient receptor potential channels (TRPs) are a very important calcium channel family [30,31,32]. They can be classified into seven subgroups, depending on different structural elements and functions. TRPs are typically expressed in excitable cells but also in non-excitable cells such as corneal and conjunctiva cells (review [33]). TRPs can be activated by different stimuli such as temperature, chemicals, tension, or crosstalk with other receptors. They serve, for instance, as temperature and nociceptor sensors and stimulate immunological reactions through modulation of calcium regulation [34,35]. The TRPV1 (vanilloid 1), known as the capsaicin receptor [36], and the TRPM8 (melastatin 8), known as the menthol receptor [37,38] belong to the temperature-sensitive TRPs (thermo-TRPs). They are functionally expressed in human conjunctival epithelial cells (HCjEC) [39,40]. 

TRPV1 is activated by heat (>43 °C), hyperosmolarity, or acidification [41,42,43,44]. In peripheral nerve fibers, TRPV1 modulates nociceptive effects, while in non-neural tissue, it is part of infectious and allergic reactions [44,45,46,47]. The TRPV1 channel induces Ca^2+^-dependent apoptosis [48], but it is also associated with wound healing and cellular proliferation in corneal tissue [49]. TRPV1 overexpression might also play a role in pterygium pathogenesis [50,51,52]. Especially in allergic conjunctivitis, TRPV1 activates sensory neurons, causing pain and pruritus [53,54].

TRPM8 is a cold receptor (<30 °C) and can be partially voltage-dependent [43,55]. Its activation induces cold pain after inflammation, for example, after conjunctivitis [56,57]. TRPM8 interacts with TRPV1 to affect anti-inflammatory responses [34]. Like TRPV1, TRPM8 is expressed at higher levels in pterygia cells [51]. Moreover, TRPM8 activation is involved in upregulating tear film production to ensure sufficient lubrication of the ocular surface [58,59] and it contributes to the control of lipogenesis in human meibomian gland cells [60].

We describe here the roles of TRPV1 and TRPM8 in mediating physiological and immunosuppressive responses to MR and C5a. The results indicate that even though the responses to these mediators are opposite to one another, they are both mediated through TRPV1 and TRPM8 activation. This codependence on TRPV1 and TRPM8 activation suggests that the responses that they elicit are mediated by different cell downstream signaling pathways.

## 2. Materials and Methods

### 2.1. Materials

Cell culture medium and other cell culture supplements were purchased from Biochrom AG (Berlin, Germany) or GIBCO Invitrogen (Karlsruhe, Germany). C5a and C5L2p were purchased from Hycult Biotech (Uden, The Netherlands) [61]. *Mucor racemosus e voluminae cellulae* was used in this study, which was kindly provided by Dr. Sonntag (SANUM-Kehlbeck GmbH & Co. KG; Hoya, Germany). This extract is a consistent, characterized substance that the company Sanum-Kehbeck uses as an active ingredient in the drug Mucokehl [62]. AMG 9810 and AMTB were purchased from Cayman Chemical Company (Ann Arbor, MI, USA). La^3+^ was purchased from Sigma-Aldrich (St. Louis, MO, USA). Calcium-imaging fura2/AM was ordered from PromoCell (Heidelberg, Germany).

### 2.2. Cell Culture of HCjEC

The spontaneously immortalized and established IOBA-NHC-cell line developed by Y. Diebold et al. in 2003 (Valladolid, Spain) was used for all experiments as a representative HCjEC model (kindly provided by Friedrich Paulsen and Fabian Garreis, Erlangen, Germany) [63]. The cells were cultivated at 37 °C with 5% CO_2_ in 1:1 DMEM/HAM’s F-12 (Gibco BRL^®^ Invitrogen) supplemented with mouse EGF (2 ng/mL), bovine insulin (1 µg/mL), cholera toxin (0.1 µg/mL), hydrocortisone (5 µg/mL) (all Sigma), and heat-inactivated fetal bovine serum (EC approved) (10%). A penicillin/streptomycin mixture (5000 units/mL) was used. Medium (4 mL) was changed every 2 to 3 days. Flasks (25 cm^2^) with filter flaps are from Nunc^TM^ (Life Technologies GmbH, 64,293 Darmstadt, Germany) [39,63].

### 2.3. Fluorescence Calcium Imaging

Fluorescence calcium imaging is a fluorescence optical method to detect very small changes in intracellular Ca^2+^ concentration in single cells [64]. For calcium imaging, the cells were seeded on coverslips (ø: 15 mm) in a 12-well plate. The cells were measured at 50–90% density and pre-incubated in cell culture medium with 1 µM fura-2/AM [64] in a dark chamber at 37 °C. After 20–40 min, they were washed in Ringer-like solution (RLS) containing 150 mM NaCl, 6 mM CsCl, 1.5 mM CaCl, 1 mM MgCl_2_, 10 mM glucose, and 10 mM HEPES at 317 mOsmol/L and pH 7.4. The 10 min measurements were performed at constant room temperature (22 °C) in a 2 mL bath chamber filled with RLS as a control. After 4 min, the drug dissolved in RLS was added. For the blocker experiments, HCjEC were additionally pre-incubated in RLS together with fura-2/AM containing the blocker for approx. 30–40 min [65,66]. The photometry setup contained a fluorescence microscope (Olympus BW50WI, Europa Holding GmbH, 20,097 Hamburg, Germany), equipped with a Omikron V. 1.0 software-controlled high-powered fluorescence LED light source (LED-Hub by Omikron, Rodgau–Dudenhoven, Germany) as well as a black and white digital camera (Olympus XM10, Olympus, Hamburg, Germany), which was connected with the U-RTC-Real-Time Controller (Olympus Europa Holding GmbH, Hamburg, Germany) with a computer and controlled over cellSens Dimension V. 1.16 software (Olympus Europa Holding GmbH, Hamburg, Germany). During the measurements, cells were exposed alternately to light at the 340 nm (2.8 s) and 380 nm (920 ms) wavelengths specified for fura-2/AM. The corresponding emission wavelength was 510 nm (green fluorescence light) [64]. In a selected section, the cells and a non-fluorescing background were marked as regions of interest (ROI) with cellSens software. The fluorescence-ratio (f_340_/f_380_) proportional to intracellular calcium concentration was calculated by the cellSens software in different cell types [64,67,68]. For evaluation, the fluorescence ratios were normalized (control set to 0.1) and drift corrected with TIDA software V. 5.25 for Windows (HEKA Electronic, Lamprecht, Germany) [39,66,67]. The averaged data with n-values indicating the number of experiments are presented as mean traces of f_340_/f_380_ ± SEM (error bars in two directions).

### 2.4. Planar Patch-Clamp Recording

The planar patch-clamp technique was used to record whole-cell currents. The measurements were performed as previously described [69]. In brief, one single cell was sucked into the 1–3 µm aperture of a microchip [70] and covered on the inside with an intracellular-like solution. The internal solution inside the chip contained 50 mM CsCl, 10 mM NaCl, 60 mM CsF, 20 mM EGTA, and 10 mM HEPES at pH 7.2 and 288 mOsmol. The external solution contained 140 mM NaCl, 4 mM KCl, 1 mM MgCl_2_, 2 mM CaCl_2_, 5 mM D-glucose-monohydrate, and 10 mM HEPES at pH 7.4 and 298 mOsmol. Both solutions were provided by Nanion Technologies GmbH (Nanion^®^, Munich, Germany). Using a software-controlled pump, a cell could be fixed into the aperture. After sealing, a break into the whole-cell configuration was made for the whole-cell current recordings [71,72]. Necessary compensations of series resistances and fast and slow capacitance transients were operated by the EPC-10 amplifier along with the PatchMaster software (Version 2.73 from HEKA Electronic, Lamprecht/Pfalz, Germany). Mean membrane capacitance (9.97 ± 0.30 pF; *n* = 59) and mean access resistance (8.66 ± 1.42 MΩ; *n* = 59) were determined by the PatchMaster software. The liquid junction potential was determined and considered after Barry [73]. All experiments were performed at ≈21 °C, room temperature, unless stated otherwise. The holding potential (HP) was set to 0 mV to prevent voltage-dependent Ca^2+^-channel activity. Every 5 s, the voltage stimulation without steps went from −60 mV (maximum inward currents) to 130 mV (maximum outward currents) for 500 ms. After 20 voltage stimulations (100 s), the drug was applied. Notably, the HCjEC are small, fragile cells that are difficult to patch. A limitation of these measurements was that leak currents and access resistance partially fluctuated during the recordings (see limitations). In many cases, the recordings had to be briefly interrupted for new compensation (see limitations). Current density (pA/pF) was calculated by normalizing currents using cell membrane capacitance [69,71].

### 2.5. Statistical Data Analyses

Paired data were probed for normality, according to Kolmogorov-Smirnov. The Student’s *t*-test assessed the statistical significance of paired data if they passed normality. If the normality test failed, the Wilcoxon matched pairs test was instead used. Likewise, statistical significance was determined for unpaired data using the Student’s *t*-test if they passed normality testing or the Mann-Whitney-U test if they failed normality testing. Probabilities of *p* < 0.05 (indicated by asterisks for paired data (*) and hash tags (#) for unpaired data) were considered significant. Statistical tests were performed, and diagrams were created using SigmaPlot version 12.5 for Windows (Systat Software, Inc., Point Richmond, CA, USA) as well as GraphPad Prism software version 5.00 for Windows (La Jolla, CA, USA). The number of replicates is shown in each case in brackets, near the traces or bars. All values are given as means ± standard error of the mean (SEM) (error bars in both directions).

## 3. Results

### 3.1. C5a Increases Intracellular Ca^2+^

**Figure 1:**
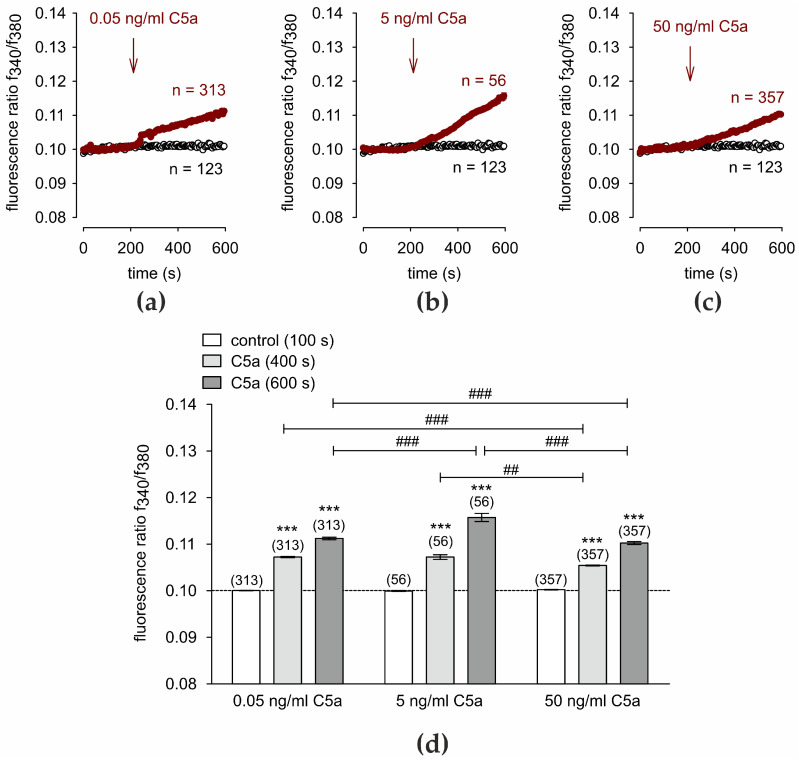
C5a increases the Ca^2+^-concentration in HCjEC at 0.05, 5, and 50 ng/mL. Data are means ± SEM. The reagents are added at the time points indicated by arrows. A control baseline measurement shows invariant Ca^2+^ levels (*n* = 123; open circles). (**a**) 0.05 ng/mL C5a induces an irreversible increase in intracellular Ca^2+^-concentration (*n* = 313, red-filled circles). (**b**) Same experiment as shown in (**a**), but with 5 ng/mL C5a (*n* = 56, red-filled circles). (**c**) In the presence of 50 ng/mL C5a, Ca^2+^ similarly increases (*n* = 357, red-filled circles). (**d**) Summary of the experiments with C5a in HCjEC. The dashed line is the reference line at 0.1. The asterisks (***) designate significant increases in [Ca^2+^]_i_ with C5a (*t* = 400 s, 600 s; *n* = 56–357; *p* < 0.001; paired tested) (gray bars) compared to controls (white bars) (*t* = 100 s). The hashtags (###) indicate statistically significant differences between C5a at different concentrations (0.05 ng/mL, 5 ng/mL, 50 ng/mL) (*t* = 400 s, 600 s; *n* = 56–357; ## *p* < 0.01; ### *p* < 0.001; unpaired tested).

C5a was applied at different concentrations (0.5 ng/mL, 5 ng/mL, and 50 ng/mL) after Ca^2+^ baseline recording (control) (4 min). Large irreversible increases in the fluorescence ratio were induced at all concentrations (Figure 1).

C5a at 0.05 ng/mL increased the fluorescence ratio f_340_/f_380_ from 0.1000 ± 0.00005 (*t* = 100 s) to 0.10720 ± 0.00015277 (*t* = 400 s) and 0.1112 ± 0.00029 (*t* = 600 s; *n* = 313; *p* < 0.0001) (Figure 1a,d). With 5 ng/mL C5a, the fluorescence ratio increased from 0.0999 ± 0.0001 (*t* = 100 s) (control) to 0.10720 ± 0.0005 (*t* = 400 s) and 0.1157 ± 0.00087 (*t* = 600 s) (*p* = 56; *n* < 0.001; paired tested) (Figure 1b,d). An increase from 0.1002 ± 0.000059 (*t* = 100 s) (control) to 0.1054 ± 0.0001 (400 s) and 0.1102 ± 0.0003 (*t* = 600 s) was measured using 50 ng/mL C5a (*n* = 357; *p* < 0.001) (Figure 1c,d). Notably, 5 ng/mL C5a provoked the largest Ca^2+^ increase after 600 s (*p* < 0.001).

### 3.2. TRP Channel Blockers Abolish C5a-Induced Ca^2+^ Increase

**Figure 2:**
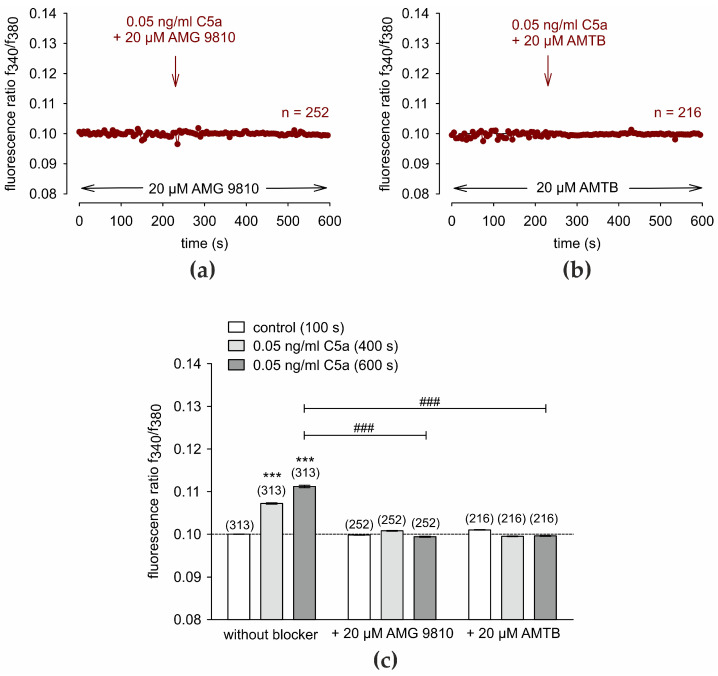
TRP channel blockers block the C5a-induced Ca^2+^ increase. Data are means ± SEM. The reagents are added at the time points indicated by arrows. A control baseline is measured, showing an invariant Ca^2+^ baseline (4 min) in the presence of the TRP channel blockers (*n* = 216–252). (**a**) Same experiment as shown in Figure 1a, but in the presence of AMG 9810 (20 µM) (*n* = 252). (**b**) AMTB (20 µM) had a similar inhibitory effect (*n* = 216). (**c**) Summary of the experiments with C5a and the TRP channel blockers in HCjEC. The dashed line is the reference line at 0.1. The asterisks (***) designate significant increases in [Ca^2+^]_i_ without blocker (*t* = 400 s, 600 s; *n* = 313; *p* < 0.001; paired tested) (gray bars) compared to controls (white bars) (*t* = 100 s). The hashtags (###) indicate statistically significant differences between C5a with and without the aforementioned TRP channel blockers (*t* = 400 s, 600 s; *n* = 216–313; *p* < 0.001; unpaired tested).

TRP-channel activation by C5a at a concentration of 0.05 ng/mL was tested in the presence of the TRPV1 blocker AMG 9810 [74] and the TRPM8 blocker AMTB [58,75] (both 20 µM). Both channel blockers abolished the C5a-induced Ca^2+^ increase (Figure 2).

Specifically, AMG 9810 reduced intracellular Ca^2+^ to near baseline levels of 0.09944 ± 0.0001 compared to the C5a positive control experiment shown in Figure 1a. (*t* = 600 s; *n* = 252; *p* < 0.001; unpaired tested). AMG 9810 and AMTB completely blocked rises in Ca^2+^ induced by exposure to C5a (*n* = 216; *p* < 0.001) (Figure 2a–c).

### 3.3. C5L2p Suppresses the C5a-Induced Ca^2+^ Increase in a Dose-Dependent Matter

**Figure 3:**
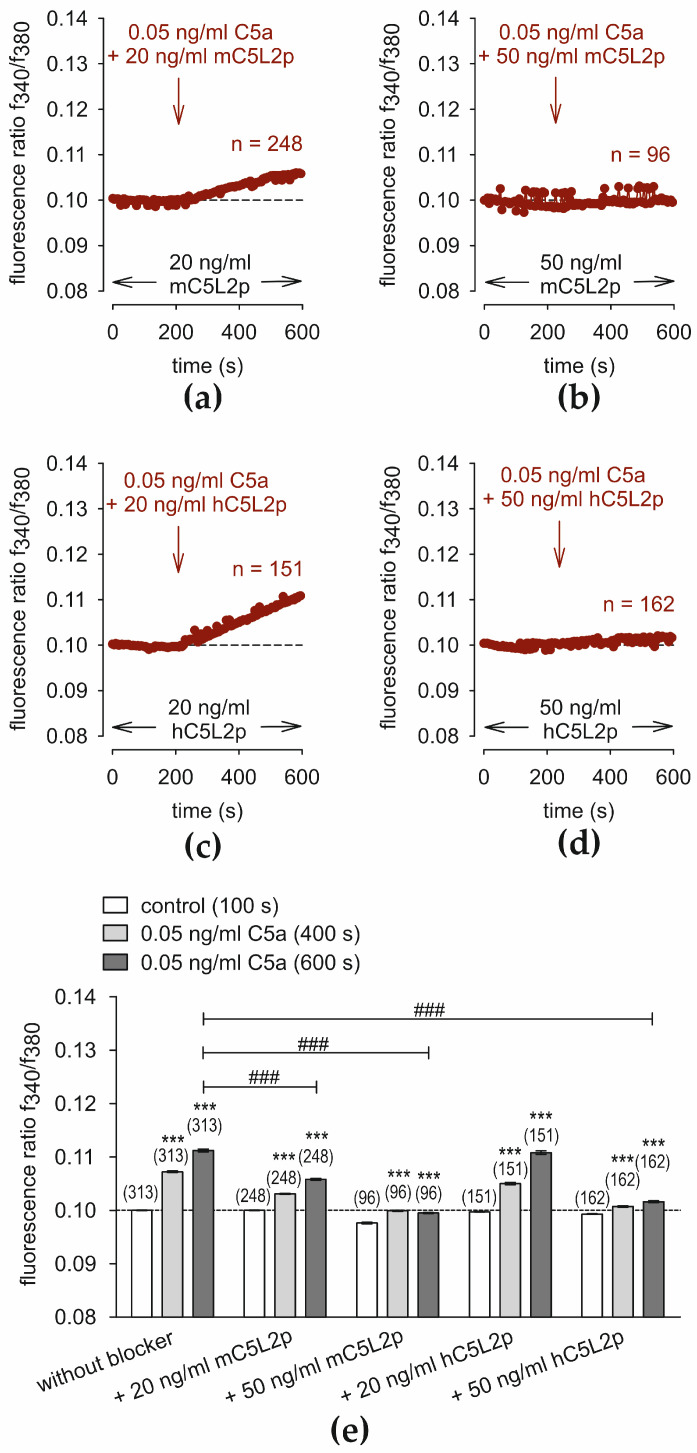
The N-terminal peptide fragment of C5L2 from human (h) of murine (m) origin (h/mC5L2p) suppresses the C5a-induced Ca^2+^ increase in a dose-dependent manner. Data are means ± SEM. The reagents are added at the time points indicated by arrows. A control baseline was measured showing Ca^2+^ invariance (4 min) in the presence of mC5L2p or hC5L2p (*n* = 96–313). (**a**) Same experiment as shown in Figure 1a, but in the presence of 20 ng/mL mouse C5L2p (*n* = 248). (**b**) 50 ng/mL mC5L2p abolishes the C5a-induced Ca^2+^-increase (*n* = 96). (**c**) 20 ng/mL hC5L2p (20 ng/mL) partially suppresses C5a-induced Ca^2+^-increase (*n* = 151). (**d**) 50 ng/mL human hC5L2p (50 ng/mL) abolishes the C5a-induced Ca^2+^-increase (*n* = 162). (**e**) Summary of the experiments with C5a and m/hC5L2p in HCjEC. The dashed line is the reference line at 0.1. The asterisks (***) designate significant change in [Ca^2+^]_i_ with C5a (*t* = 600 s; *n* = 96–313; *p* < 0.001; paired tested) compared to the control (*t* = 100 s). The hashtags (###) indicate statistically significant differences between C5a with and without m/hC5L2p at concentrations of 20 ng/mL and 50 ng/mL (*t* = 600 s; *n* = 96–313; *p* < 0.001; unpaired tested).

**Figure 4:**
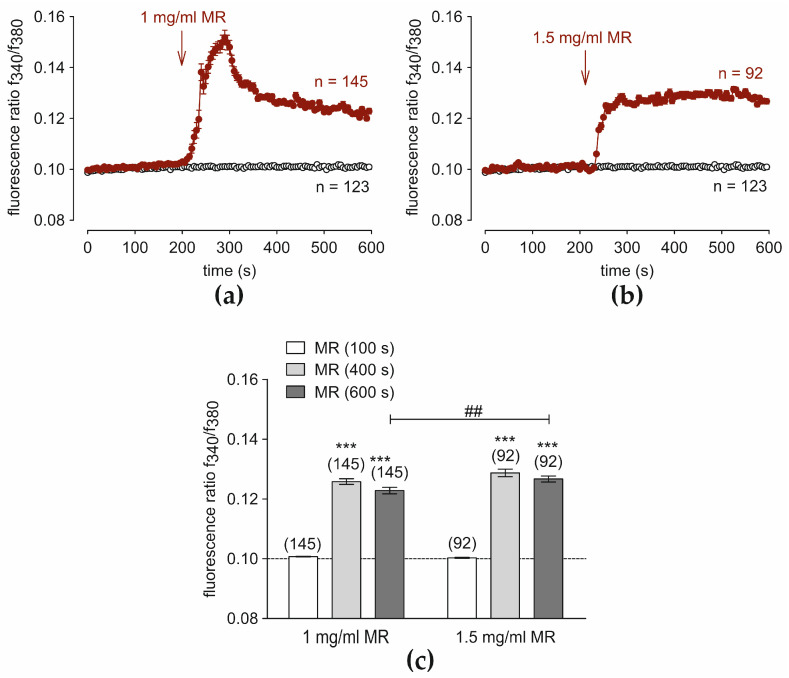
MR increases intracellular Ca^2+^. Data are means ± SEM. The reagents were added at the time points indicated by arrows. A control baseline measurement is invariant (*n* = 123; open circles). (**a**) 1 mg/mL MR induces an increase in intracellular Ca^2+^-concentration (*n* = 145, red filled circles) in HCjEC. (**b**) Same experiment as shown in (**a**), but with 1.5 ng/mL C5a (*n* = 92, red filled circles). (**c**) Summary of the experiments with MR in HCjEC. The dashed line is the reference line at 0.1. The asterisks (***) designate significant increases in [Ca^2+^]_i_ with MR (*t* = 400 s, 600 s; *n* = 92, 145; *p* < 0.001; paired tested) compared to control (*t* = 100 s). The hashtag (##) indicates statistically significant differences between MR at different concentrations of 1 mg/mL and 1.5 mg/mL); *t* = 400 s, 600 s; *n* = 92–145; *p* < 0.01; unpaired tested).

The N-terminal peptide fragment of C5L2 (C5L2p) was of mouse (m) or human (h) origin. Figure 3 shows an inhibitory effect of mC5L2p and hC5L2p on C5a-induced Ca^2+^ increase in HCjEC depending on C5L2p concentration (20 ng/mL, 50 ng/mL). In the presence of 20 ng/mL mC5L2p, the fluorescence ratio partially decreased to 0.1058 ± 0.00019 (*t* = 600 s; *n* = 248; *p* < 0.001; unpaired tested) compared to the positive control shown in Figure 1a (Figure 3a,e). In contrast, 50 ng/mL mC5L2p fully blocked the C5a effect to a fluorescence ratio of 0.0995 ± 0.0001 (*t* = 600 s; *n* = 96; *p* < 0.001; unpaired tested) (Figure 4b,e). Similar results were obtained with human C5L2p. 20 ng/mL hC5L2p partially decreased f340/f380 to 0.1108 ± 0.00032 (*t* = 600 s; *n* = 151; *p* < 0.001) compared to the positive control (Figure 1a), whereas 50 ng/mL hC5L2p fully suppressed f340/f380 to baseline levels (0.1016 ± 0.00019; *t* = 600 s; *n* = 162; *p* < 0.001; both unpaired tested) (Figure 3d,e). Taken together, 50 ng/mL mC5L2p and hC5L2p completely abolish the C5a-induced Ca^2+^ increase (Figure 3d).

### 3.4. Fungal Pathogen MR Increases Intracellular Ca^2+^

MR was used as a fungal pathogen. In the presence of extracellular Ca^2+^ in the Ringer-like solution (RLS), 1 mg/mL MR induced an initial increase in the fluorescence ratio from 0.1007 ± 0.0001 (*t* = 100 s) to 0.1518 ± 0.0029 (*t* = 290 s), followed by a decrease to 0.1258 ± 0.00095 (*t* = 400 s) and a further slight decrease to 0.1228 ± 0.0011 (*t* = 600 s; *n* = 145; *p* < 0.001; paired data) (Figure 4a,c). A similar effect resulted from exposure to 1.5 mg/mL MR, but without the transient Ca^2+^ increase at the beginning (0.1267 ± 0.001; *t* = 600 s; *n* = 92, *p* < 0.001) (Figure 4b,c). In contrast, this effect was absent in a Ca^2+^ free RLS (Figure S1). Taken together, MR increased intracellular Ca^2+^ if extracellular Ca^2+^ was present. This dependence of the rise in Ca^2+^ on the presence of extracellular Ca^2+^ suggests that Ca^2+^ channel activation at the cell membrane induced this response. 

### 3.5. TRP Channel Blockers Suppress the MR-Induced Ca^2+^ Increase

**Figure 5:**
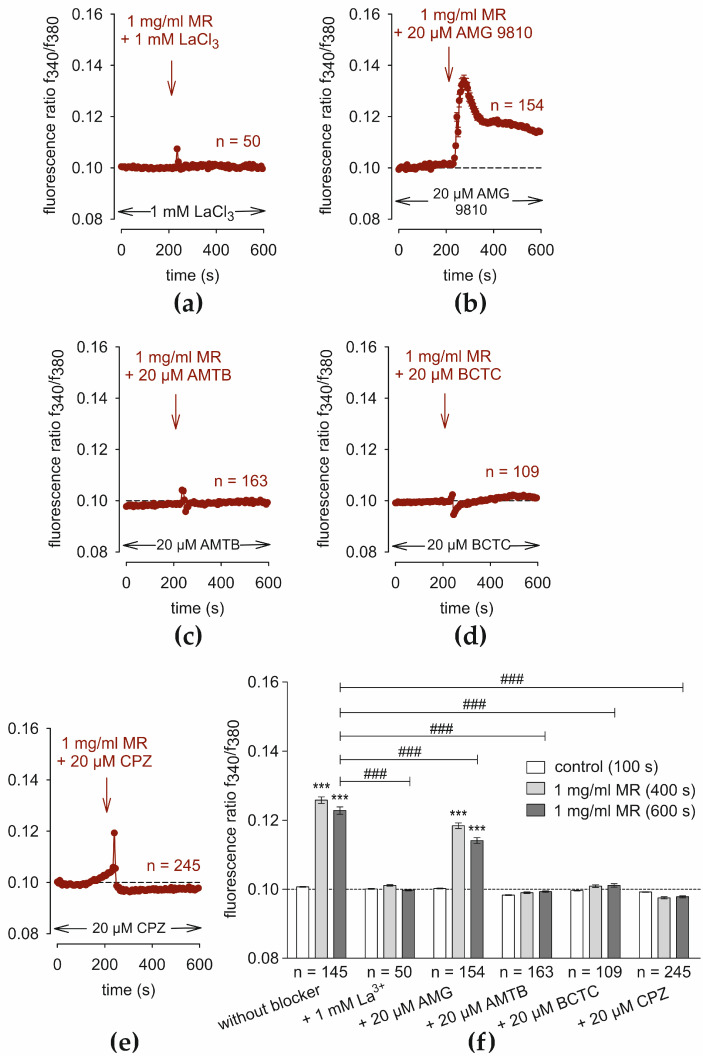
TRP channel blockers suppress the MR-induced rise in Ca^2+^. Data are means ± SEM. The reagents are added at the time points indicated by arrows. An invariant control baseline is evident (4 min) in the presence of the TRP channel blockers (*n* = 50–245). (**a**) Same experiment as shown in Figure 4a, but in the presence of La^3+^ (1 mM), which completely blocks the MR-induced Ca^2+^-increase (*n* = 50). (**b**) AMG 9810 (20 µM) only partially suppresses the MR-rise in Ca^2+^-increase (*n* = 154). (**c**) AMTB (20 µM) completely blocks the MR-rise in Ca^2+^ (*n* = 163). (**d**) Same result with BCTC (20 µM) (*n* = 109). (**e**) CPZ (20 µM) temporarily suppresses the MR-rise in Ca^2+^-increase slightly below the baseline (dashed line) (*n* = 245). (**f**) Summary of the experiments with MR and the TRP channel blockers in HCjEC. The dashed line is the reference line at 0.1. The asterisks (***) designate significant increases in [Ca^2+^]_i_ with MR (*t* = 400 s and *t* = 600 s; *n* = 50–245; *** *p* < 0.001; paired tested) relative to control (*t* = 100 s). The hashtags (###) indicate statistically significant differences between MR with and without the aforementioned TRP channel blockers (*t* = 600 s; *n* = 50–245; *p* < 0.001; unpaired tested).

The effects were determined of lanthanum-III-chloride (La^3+^) (1 mM), AMG 9810 (20 µM), AMTB (20 µM), and the TRPV1/TRPM8 channel blocker capsazepine (20 µM) [76] as well as the TRPV1/TRPM8 blocker BCTC (20 µM) [77,78] on the rise in Ca^2+^ induced by 1 mg/mL MR. At first, La^3+^ [79,80] completely abolished the MR-induced Ca^2+^ increase (0.0997 ± 0.00019; 600 s; *n* = 50; *p* < 0.001; unpaired data) compared to the Ca^2+^ response shown in Figure 4a (positive control) (Figure 5a). AMG 9810 (20 µM) had a similar Ca^2+^ response pattern (Figure 5b), but only at a slightly lower level (0.1141 ± 0.0008; *t* = 600 s; *n* = 154; *p* < 0.001; unpaired data). AMTB (20 µM) completely blocked the MR-induced Ca^2+^ increase (0.0993 ± 0.0002; *t* = 600 s; *n* = 163) (Figure 5c). In addition, BCTC (20 µM) had a similar blocking effect on MR-induced Ca^2+^-influx (0.1011 ± 0.0005; *t* = 600 s; *n* = 109; *p* < 0.001) (Figure 5d). Finally, CPZ also suppressed the fluorescence ratio, even temporarily slightly below the baseline (0.0978 ± 0.00029; *t* = 600 s; *n* = 245; *p* < 0.001) (Figure 5e). In summary, La^3+^, AMTB, BCTC, and CPZ completely blocked the MR-induced Ca^2+^ increase, whereas AMG 9810 partially inhibited this response (Figure 5f).

### 3.6. C5a Suppresses the MR-Induced Ca^2+^ Increase

**Figure 6:**
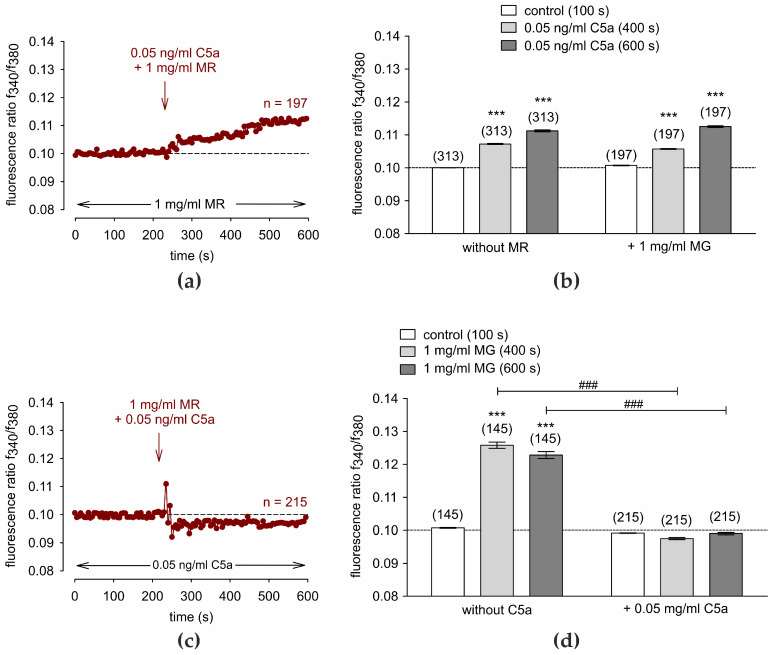
Interplay between MR and C5a. Data are means ± SEM. The reagents are added at the time points indicated by arrows. The dashed line is the reference line at 0.1. A control baseline shows a Ca^2+^ homeostasis (4 min) in the presence of MR or C5a (*n* = 197–313). (**a**) Same experiment as shown in Figure 1a, but in the presence of 1 mg/mL MR. 0.05 ng/mL C5a increases the intracellular Ca^2+^ (*n* = 197). (**b**) Summary of the experiments with C5a and MR in HCjEC. The asterisks (***) designate significant increases in [Ca^2+^]_i_ with C5a (*t* = 600 s; *n* = 197; *** *p* < 0.001; paired tested) compared to control (*t* = 100 s). (**c**) 0.05 ng/mL C5a completely blocked the MR-induced Ca^2+^-increase to just below the baseline (dashed line; *n* = 215). (**d**) Similar analysis as shown in (**b**), but with C5a preincubation instead of MR. The asterisks (***) designate significant increases in [Ca^2+^]_i_ with C5a (*t* = 600 s; *n* = 145; *** *p* < 0.001; paired tested) compared to control (*t* = 100 s). The hashtags (###) indicate statistically significant differences between MR with and without C5a (*t* = 600 s; *n* = 50–245; *p* < 0.001; unpaired tested).

Figure 6a shows that C5a (0.05 ng/mL) induced a similar Ca^2+^ response pattern irrespective of the presence or absence of MR preincubation (*n* = 197, *p* < 0.001, paired tested, Figure 6b). In contrast, the MR-induced Ca^2+^ increase in the presence of 0.05 ng/mL C5a was completely blocked (*n* = 215; *p* < 0.001, unpaired tested) (Figure 6c,d). Therefore, the anaphylatoxin C5a blocks a Ca^2+^ overload through suppression of a fungal pathogen-induced Ca^2+^ increase.

### 3.7. C5a-Increased Whole-Cell Currents Are Suppressed by TRP Channel Blockers

**Figure 7:**
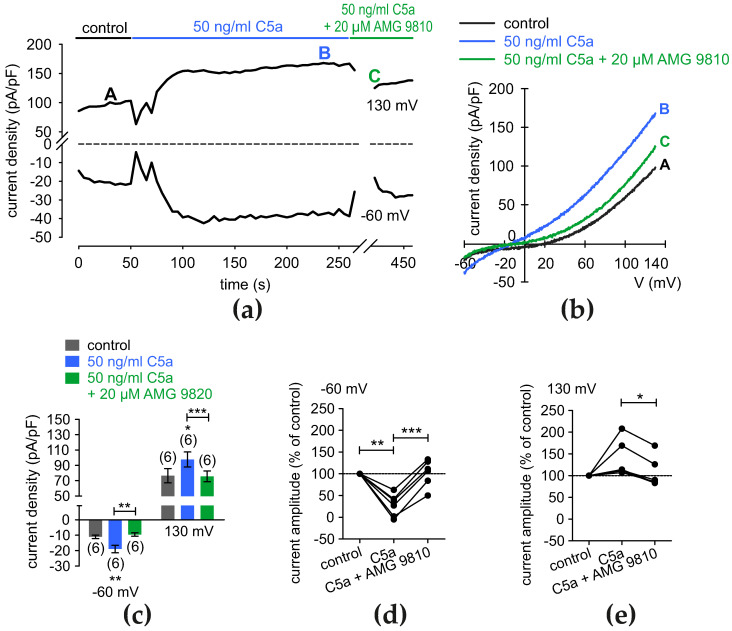
C5a increases whole-cell currents through increases in TRPV1 activity. (**a**) Time-course recording of the increases in the current induced by C5a (50 ng/mL) that are suppressed by AMG 9810 (20 µM). The dashed line is the reference line at 0 pA/pF. (**b**) Original traces of current changes induced by voltage ramps in the presence of C5a. Current densities are shown before application as control (labeled as A), during application of 50 ng/mL C5a (labeled as B), and after addition of 20 µM AMG 9810 (labeled as C). (**c**) Summary of the experiments with C5a and AMG 9810. The asterisks (*) indicate statistically significant differences of whole-cell currents with and without C5a (*n* = 6; * *p* < 0.05, ** *p* < 0.01; paired tested) and significant differences of C5a-induced rises with and without AMG 9810 (*n* = 6; ** *p* < 0.01, *** *p* < 0.005; paired tested). (**d**) Maximum negative current amplitudes that a voltage step from 0 to −60 mV induces are shown as percent of control values before application of 50 ng/mL C5a (control set to 100%; dashed line). C5a-induces rises in inward currents that 20 µM AMG 9810 suppresses. (**e**) Same diagram as shown in (**d**), but related to the maximum positive current amplitudes that a voltage step from 0 to +130 mV induces. AMG 9810. (20 µM) suppresses C5a-rises in outward currents.

Earlier studies by Fan et al. were the first to demonstrate the effects of 5 nM C5a on intracellular Ca^2+^ and the rapid triggering of outwardly directed rectifying K^+^ conductance in murine monocytes [81]. By measuring whole-cell currents, we tested whether the anaphylatoxin C5a activates non-selective cation channels. Significant increases in the whole-cell currents were induced by a voltage ramp from −60 mV to 130 mV (Figure 7a). The maximum inward currents at −60 mV increased towards control currents from −11.07 ± 1.15 pA/pF to −18.97 ± 2.43 pA/pF (*p* < 0.001; *n* = 6; Figure 7a–c). AMG 9810, as a specific TRPV1 blocker [74], suppressed C5a-induced increases in whole-cell currents to −9.57 ± 1.08 pA/pF (*p* < 0.01; *n* = 6; Figure 7c). At 130 mV, C5a increased the outward currents from 76.55 ± 9.27 pA/pF to 97.73 ± 9.86 pA/pF (*p* < 0.05; *n* = 6), and these rises were completely suppressed by AMG 9810 to 75.63 ± 7.01 pA/pF (*p* < 0.001; *n* = 6) (Figure 7c). Furthermore, C5a increased the maximum inward current amplitudes induced by a voltage step from 0 mV to −60 mV from control (set to 100%) to 28 ± 11% (*p* < 0.01; *n* = 6), while this rise was reduced to 102 ± 13% (*p* < 0.001; *n* = 6), by AMG 9810 (Figure 7d). Similarly, maximum outward current amplitudes induced by a ramp from 0 mV to 130 mV rose to 137 ± 17% from control by application of C5a, and AMG 9810 decreased them to 106 ± 14% (*p* < 0.05; *n* = 6) by Figure 7e. 

Similar results were obtained using AMTB [82]. Maximum inward currents at −60 mV rose from −19.64 ± 3.83 pA/pF to −77.71 ± 17.27 pA/pF (*p* < 0.01; *n* = 8) and maximum outward currents at 130 mV increased from 262 ± 39.94 pA/pF to 435.9 ± 67.92 pA/pF (*p* < 0.01; *n* = 8) (Figure S2). AMTB reduced the inward current densities to −26.85 ± 7.20 pA/pF (*p* < 0.01; *n* = 8), and the outward current densities were suppressed to 328.70 ± 60.47 pA/pF (*p* < 0.01; *n* = 8) (Figure S2). Taken together, C5a-induces a rise in the whole-cell currents through activating both TRPV1 and TRPM8 channels.

### 3.8. MR Increases Whole-Cell Currents

**Figure 8:**
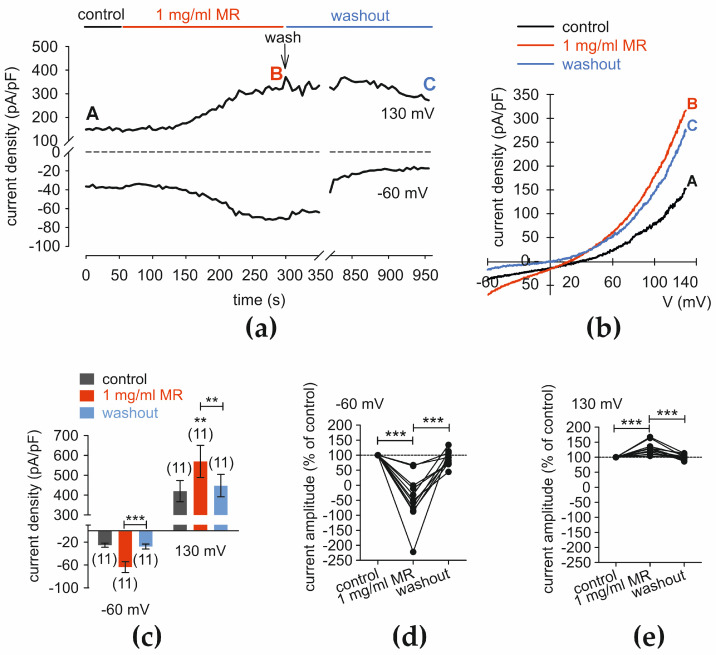
MR increases whole-cell currents. (**a**) Time course recording of the current increases that MR induces (1 mg/mL) and declines that occur after replacement with fresh solution. The dashed line is the reference line at 0 pA/pF. (**b**) Original traces of MR-current rises that occur due to voltage ramps. Current densities are shown before application as a control (labeled as A), during application of 1 mg/mL MR (labeled as B), and after washout (labeled as C). (**c**) Summary of planar patch-clamp experiments with MR. The asterisks (**) indicate statistically significant differences in whole-cell currents with and without MR (*n* = 11; *p* < 0.01; paired tested). (**d**) Maximum negative current amplitudes that a voltage step from 0 to −60 mV creates are shown as percent of control values before application of 1 mg/mL MR (control set to 100%; dashed line). MR induces rises in inward currents that decline to a control level after washout. The asterisks (***) indicate statistically significant differences in whole-cell currents with and without MR (*n* = 11; *p* < 0.001; paired tested). (**e**) Same diagram as shown in (**d**), but show the maximum rises in positive current amplitudes that a voltage step from 0 to +130 mV induces. MR also induces a large increase in outward currents.

A study by Wang et al. revealed that a putative TRP-like calcium channel (trpR) in the filamentous fungus *Aspergillus nidulans* performs important roles in conidiation and in adapting to cell wall disruption reagents. It was suggested that TrpR functions as a Golgi membrane calcium ion channel to mediate cell wall integration [83]. As already shown in this study, the ubiquitarian fungus MR triggered a Ca^2+^ influx in the presence of Ca^2+^ (Figure S1). This shows that this response to trpR is due to the activation of a plasma membrane pathway. Such a change is consistent with a large MR-induced increase in whole-cell currents in HCjEC (Figure 8).

At −60 mV, MR increased whole-cell inward currents from −25.64 ± 3.61 pA/pF to −58.56 ± 8.05 pA/pF (*p* < 0.001; *n* = 11) and maximum outward currents at 130 mV from 442.1 ± 53.9 pA/pF to 585.2 ± 86.46 pA/pF (*p* < 0.01; *n* = 11) (Figure 8a–c). After washout, the inward currents fell to −27.78 ± 4.36 pA/pF (*p* = 0.01; *n* = 11), and the outward currents decreased to 447.40 ± 56.54 pA/pF (*p* = 0.01; n = 11) (Figure 8a–c). If control currents were normalized to 100%, the amplitude of maximum inward currents (at −60 mV) increased to −45 ± 24% and decreased after washout to 90 ± 8% (both *p* < 0.001; *n* = 11) (Figure 8d). Similarly, maximum outward current amplitude rose to 129 ± 6% after MR application and reversed back to 100 ± 3% after its washout (both *p* < 0.001; *n* = 11) (Figure 8e).

### 3.9. MR-Induces Rises in Whole-Cell Currents That TRP Channel Blockers Suppress

**Figure 9:**
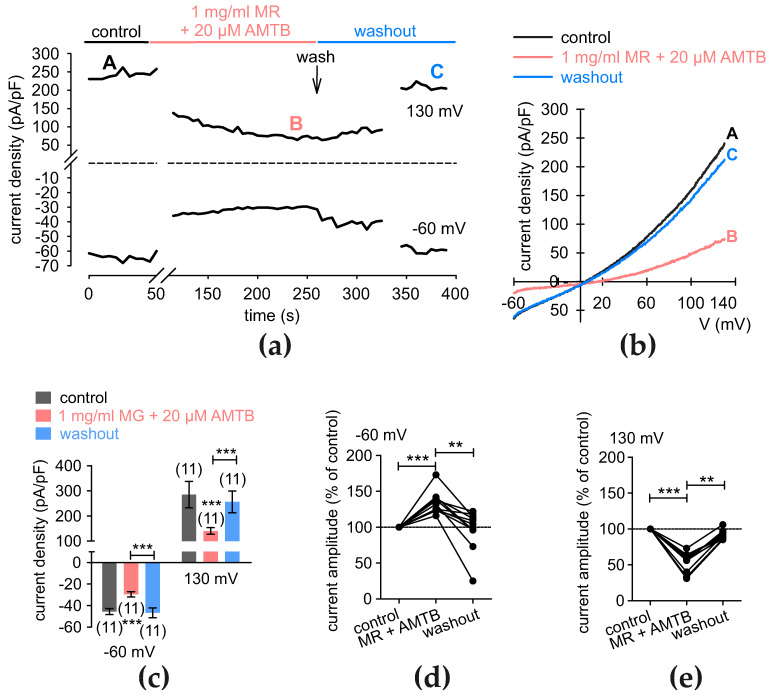
MR increases whole-cell currents through TRPM8 activation. (**a**) Time course recording of the current increases that MR (1 mg/mL) induces and AMTB (20 µM) inhibits. The dashed line is the reference line at 0 pA/pF. (**b**) Original traces of rises in currents that MR augments of rises initially induced by voltage ramps. Current densities are shown before application as control (labeled as A), during application of 1 mg/mL MR (labeled as B), and after addition of 20 µM AMTB (labeled as C). (**c**) Summary of planar patch-clamp experiments with MR + AMTB. The asterisks (***) indicate statistically significant differences in whole-cell currents with and without MR + AMTB (*n* = 11; *** *p* < 0.001; paired tested). (**d**) Maximum negative current amplitudes that a voltage step from 0 to −60 mV induces are shown as percent of control values before application of 1 mg/mL MR + 20 µM AMTB (control set to 100%; dashed line). MR + 20 µM AMTB reduces inward currents below control and washout levels. The asterisks (**) and (***) indicate statistically significant differences in whole-cell currents with and without MR + AMTB (*n* = 11; ** *p* < 0.01, *** *p* < 0.001; paired tested). (**e**) Same diagram as shown in (**d**), but shown as maximum positive current amplitudes induced by a voltage step from 0 to +130 mV.

As in the C5a protocol, the individual effects were determined of AMG 9810 and AMTB on the MR-induced increases in the whole-cell currents. AMTB decreased whole-cell inward currents from −45.55 ± 2.79 pA/pF to −29.56 ± 2.52 pA/pF (*p* < 0.001; *n* = 11). After washout, the currents recovered to −46.73 ± 4.58 pA/pF (*p* = 0.001; *n* = 11) (Figure 9a–c). Similarly, maximum outward currents decreased after application of AMTB from 284.8 ± 52.66 pA/pF to 140.50 ± 13.11 pA/pF. After washout, the currents rose to 255.70 ± 43.32 pA/pF (both *p* < 0.001; *n* = 11) (Figure 9a–c). If the currents were normalized to 100%, AMTB decreased maximum inward current amplitudes at −60 mV to 134 ± 5% (*p* < 0.001; *n* = 11) and reversed back after washout to 97 ± 8% (*p* < 0.01; *n* = 11) (Figure 9d). Similarly, the maximum outward current amplitudes at 130 mV decreased to 54 ± 4% (*p* < 0.001, *n* = 11) from the control by application of MR and AMTB and increased back to 91 ± 2% after washout (*p* = 0.01; *n* = 11) (Figure 9e).

Similar results were obtained with AMG 9810 (20 µM) (Figure S3). In brief, maximum inward current densities decreased from −39.82 ± 1.00 pA/pF to −30.11 ± 2.10 pA/pF and increased after washout to 41.48 ± 2.80 pA/pF (both *p* < 0.01; *n* = 9). At 130 mV, the maximum outward currents changed from 212 ± 5.76 pA/pF to 141.3 ± 6.99 pA/pF and 219.7 ± 9.54 pA/pF after washout (both *p* = 0.001; *n* = 9) (Figure S3a–c). 

### 3.10. C5a Suppresses the Whole Cell Current Rises That MR Induces

**Figure 10:**
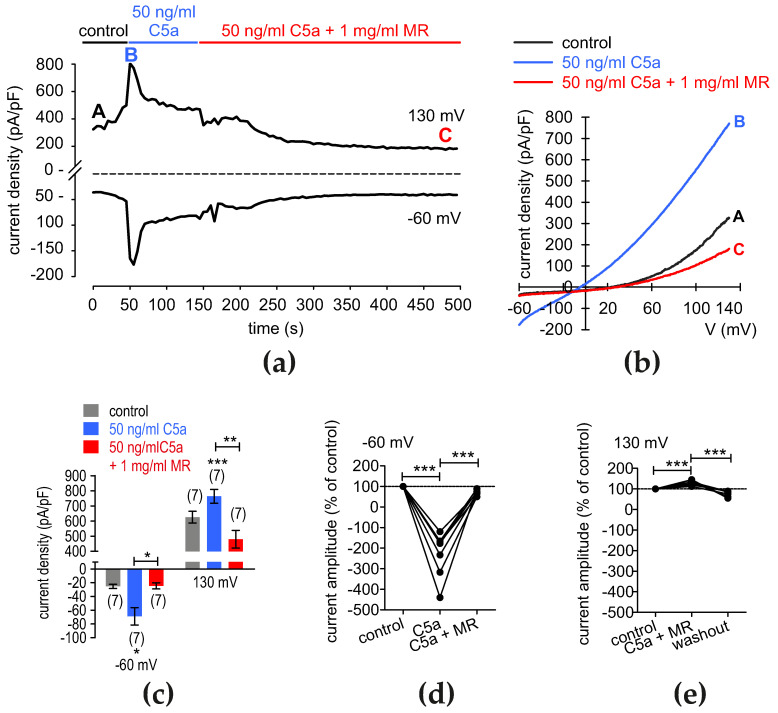
C5a suppresses MR-induced rises in currents. (**a**) Time course recording of the current increases that C5a (50 ng/mL) induces and decreases that result from the application of MR (1 mg/mL). The dashed line is the reference line at 0 pA/pF. (**b**) Original traces of C5a and rises in currents that MR-induces exceed those that a voltage creates. Current densities are shown before application as control (labelled as A), during application of 50 ng/mL C5a (labeled as B), and after addition of 1 mg/mL MR (labeled as C). (**c**) Summary of the experiments with C5a + MR. The asterisks (*), (**) and (***) indicate statistically significant differences in whole-cell currents with and without C5a and following the addition of MR (*n* = 7; * *p* < 0.05, ** *p* < 0.01, *** *p* < 0.001; paired tested). (**d**) Maximum negative current amplitudes at −60 mV are shown as percent of control values before application of 50 ng/mL C5a (control set to 100%; dashed line). C5a increases inward currents, which decline in the presence of MR (*n* = 7; *p* < 0.001). The asterisks (***) indicate statistically significant differences in whole-cell currents with and without C5a and following the addition of MR (*n* = 7; *** *p* < 0.001; paired tested. (**e**) Same diagram as shown in (**d**), but related to maximum outward current amplitudes at +130 mV. The outward currents undergo a similar effect (*n* = 7; *p* < 0.001), but the changes are smaller than those in the inward currents.

There is agreement between the calcium imaging and the whole-cell current results. At −60 mV, C5a increased the whole-cell currents from −25.26 ± 3.13 pA/pF to −68.86 ± 12.68 pA/pF (*p* = 0.05; *n* = 7) and maximum outward currents at −130 mV from 625.8 ± 39.25 pA/pF to 763.70 ± 45.81 pA/pF (*p* = 0.001; *n* = 7) (Figure 10a–c). MR (1 mg/mL) decreased the whole-cell currents to a final steady state (Figure 10a). Notable, maximum inward currents fell to −24.53 ± 4.44 pA/pF (*p* < 0.05; *n* = 7) and maximum outward currents declined to 480.10 ± 58.58 pA/pF (*p* < 0.01; *n* = 7) (Figure 10a–c). 

After setting the control currents to 100% at −60 mV, C5a increased the maximum inward current amplitudes to −232 ± 42%, while MR decreased the amplitude to 69 ± 6% in the presence of C5a (both *p* < 0.001; *n* = 7) (Figure 10d). The C5a application also increased the maximum outward current amplitudes at 130 mV to 130 ± 4.5%. After adding MR in the presence of C5a, the current amplitude decreased to 77 ± 5.3% (both *p* < 0.001; *n* = 7) (Figure 10e). However, when MR was applied first and then C5a, they elicited an opposite (additive) effect (Figure S4). In brief, normalized inward currents increased to −39.71 ± 2.296% after application of MR, and then C5a augmented the rise to −305 ± 42% (both *p* < 0.001; *n* = 6) (Figure S4d). C5a addition increased the outward current amplitudes to 125 ± 2.5% (*p* < 0.001; *n* = 6) in the presence of MR and to 158 ± 9% (*p* = 0.01; *n* = 6) (Figure S4e). In summary, MR inhibited the rise in currents induced by the addition of C5a. This indicates a complex interplay between C5a and MR. 

## 4. Discussion

### 4.1. Role of C5a in Mediating Calcium Regulation

C5a treatment of HCjEC increased intracellular Ca^2+^ levels in a dose-dependent manner (Figure 1), as in neurons [84] and retinal pigment epithelial (RPE) cells, as well as immune cells such as neutrophil granulocytes or macrophages [15,85,86]. The dependence of these responses on TRP subtype activation is novel because in neurons and RPE cells, the C5a-induced Ca^2+^ increases are instead associated with voltage-dependent L-type channel activation [84,86]. In contrast to neurons with a resting membrane potential (RMP) below −60 mV depending on (body) temperature (the activation threshold of TRPV4 is >34 °C) [87], the RMP averaged −40 mV in RPE cells [88]. However, L-type channels can be fully activated at approximately −70 mV. Therefore, the contribution of these channels is probably minor in non-stimulated epithelial cells because of their more positive RPM [88,89]. The same applies to other human epithelial cells (e.g., HeLa and Caco-2), where a RMP of -50 mV was measured [90]. Notably, pathogens such as enteropathogenic *Escherichia coli* (EPEC) can modulate the RMP in these cells [90]. It is unknown whether other pathogens, such as the fungus MR, also mediate a similar effect in HCjEC. Taken together, the C5a-induced Ca^2+^ increase in HCjEC is essentially due to TRP ^+^ channel activation, whereas voltage-dependent L-type channels are unlikely to play a major role, as already shown in human corneal keratocytes (HCK) [91]. 

Sodkas et al. showed that C5a-induced Ca^2+^ increases were suppressed in Orai1-deficient neutrophils [15]. Orai1 is a calcium-selective ion channel that is activated upon the depletion of internal calcium stores and is related to highly Ca^2+^-selective TRPs such as TRPV5 and TRPV6 [92]. In contrast, we found that C5a had no effect on intracellular Ca^2+^ levels in a Ca^2+^-free RLS (Figure S5), indicating that the C5a-induced Ca^2+^ entry is solely due to TRP channel activation located at the cell membrane. A large increase in Ca^2+^ occurred following supplementation of the RLS with Ca^2+^ (Figure S5). This experimental design shown in Figure S5 is also used to determine a contribution from TRPV6 channel involvement, which is activated by passive store depletion [93]. If this protocol activates an increase in Ca^2+^ influx, such a response would be indicative of activation by C5a of store-operated Ca^2+^ channels—such as TRPV6 in HCjEC. Even though such a paradigm exists in various tumor cell types [94], where C5a plays a complex role in tumor progression and the tumor microenvironment [95], our results show that C5a induces rises in Ca^2+^ influx by interacting with TRPV1 and TRPM8. This independence is evident since blockage of TRPV1 with AMG 9810 and AMTB for TRPM8 blocked C5a-induced rises in Ca^2+^. Therefore, C5a appears to mediate Ca^2+^ transients through activation of plasma membrane-delimited TRPV1 and TRPM8 activity rather than depletion of intracellular Ca^2+^ stores.

### 4.2. Mechanism of the C5a Ca^2+^ Effect with and without C5L2p

**Figure 11:**
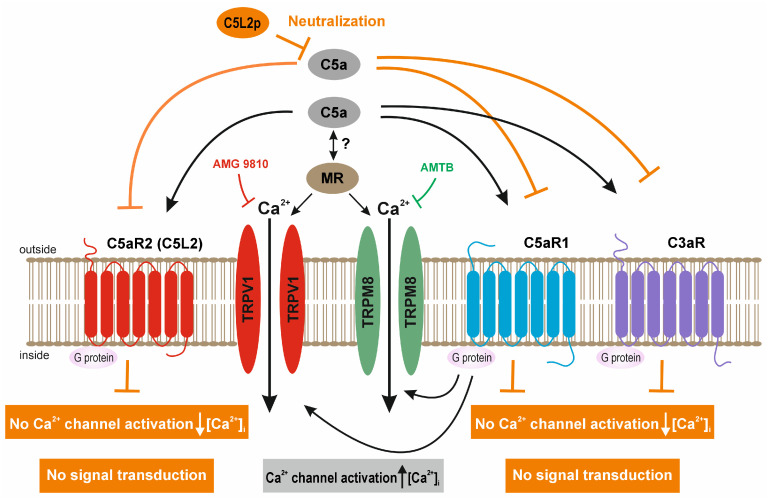
This simplified scheme accounts for how interactions between C5a, MR, and TRPV1 or TRPM8 modulate rises in Ca^2+^ influx (black arrows). Notably, C5a and MR elicit TRPV1 and TRPM8 activation in HCjEC (Figure 1, Figure 4, Figure 9 and Figure S3), whereas the interaction between C5a and MR is not fully elucidated. Ca^2+^ permeable channels such as TRPV1 can be selectively blocked by AMG 9810 [74] (red marking), and TRPM8 can be blocked by AMTB [58,75] (green marking). Two different physiological assays, such as fluorescence calcium imaging and the planar patch-clamp technique, were used to validate the effect of C5a or MR on intracellular Ca^2+^ concentration and whole-cell currents. The C5L2p used contains only the N-terminal part of the C5aR2 and serves to neutralize C5a in the extracellular compartment (orange markings). Therefore, C5L2p does not enable signal transduction [61]. The cells contain 3 GPCRs that can bind C5a (black arrows): These GPCRs are C5aR1, C5aR2 (C5L2), and C3aR. Notably, C5L2 is structurally homologous but deficient in G protein coupling [98]. Finally, C5a has no effect on Ca^2+^ regulation and no signal transduction in the presence of C5L2p neutralizing C5a (50 ng/mL) (highlighted in orange) (Figure 3).

The second C5a receptor, C5L2 (C5aR2), is often described as a decoy receptor [96]. C5L2, which is transfected into rat basophilic leukemia cells, does not support degranulation or increases in intracellular Ca^2+^ [97]. In this study, the putative inhibiting ability of the N-terminal C5L2 peptide fragment (C5L2p) [61] was examined using either the mouse or human peptide. In the presence of 20–50 ng/mL C5L2p, an inhibitory effect was clearly detectable (Figure 3), whereby the effect was even more pronounced at 50 ng/mL with a negligible difference between the mouse and human peptides. A similar result could be observed in HCK even with 50 ng/mL C5a and 20 ng/mL C5L2 [91]. One explanation could be that C5a triggers no increase in intracellular Ca^2+^ through C5L2p since this receptor fragment (protein) neutralizes C5a and does not couple to C5aR2 (C5L2), which is deficient in G protein coupling [98]. Veldhuis et al. (2015) highlighted an important association. They described a so-called GPCR-TRP axis, meaning that GPCRs can activate TRPs (and vice versa) [99]. Extracellular application of C5a activates TRPV1 and TRPM8, followed by an increase in intracellular Ca^2+^. This is not the case with C5L2p since it does not enable signal transduction [61]. Due to C5a neutralization by C5L2p, the three GPCRs (C5aR1, C5aR2/C5L2, and C3aR) cannot activate TRPs. As a result, the Ca^2+^ effect of C5a on intracellular Ca^2+^ is blocked in the presence of 50 ng/mL human or mouse C5L2p (Figure 3 and orange highlighted in Figure 11).

### 4.3. MR Affects Calcium Regulation

It is known that some toxins in human pathogenic fungi, such as *Candida albicans,* induce an increase in inward currents in human epithelial cells with a detected Ca^2+^-influx [100]. It was also described as an interaction between host cells and the fungal surface, including an increase in intracellular calcium or depolarization in phagocytes [101]. The conjunctiva, when exposed to various pathogens, can come into contact with fungal pathogens such as MR. It was in vitro electro-physiologically demonstrated in HCjEC that extracellular application of MR increased Ca^2+^-influx (Figure 4a). A fast-initial increase with the following decrease might be a sign of a temporary strong TRP channel activation following Ca^2+^ influx. Even lower concentrations (1 mg/mL) seemed to provoke a primal stronger reaction than higher concentrations (1.5 mg/mL) with a long-term similar effect (Figure 4a vs. Figure 4b). Notably, the Ca^2+^ increase could only be observed in the presence of 1.5 mM Ca^2+^ in the RLS but not in a Ca^2+^-free RLS (Figure S1). This indicates that instead of Ca^2+^ channels at the cell membrane, intracellular Ca^2+^-releasing channels are instead involved in the MR-induced Ca^2+^ increase. Overall, this experiment demonstrates that after a long application of MR without external Ca^2+^, Ca^2+^-homeostasis fluctuated around the baseline. A minor Ca^2+^ influx was barely detectable (Figure S1). Despite statistical significance, this minor Ca^2+^ increase is not comparable with the large MR-induced Ca^2+^ increase in the control experiment with 1.5 mM external Ca^2+^ in the RLS (Figure 4b).

### 4.4. MR Is Associated with TRPs

The large inhibitory effect of La^3+^ on C5a-induced rises in Ca^2+^ influx provided the first indication that increases in plasma membrane Ca^2+^ channel activity underlie this response [102] (Figure 5a). To specify which TRP channel was activated by MR, the experiments were repeated in the presence of the same TRP channel blockers for TRPV1 or TRPM8. In addition, BCTC [78,103] and CPZ [104,105,106,107] were used as TRPV1/TRPM8 blockers. Except for AMG 9810, all blockers showed a clear inhibitory effect on MR-induced Ca^2+^ influx. The smaller AMG 9810 effect may be due to its activation of multiple other (unknown) TRPs besides TRPV1. In contrast, AMTB provoked a significantly larger blocking effect (Figure 5c). This might be caused by a high functional TRPM8 expression in HCjEC, which should be further analyzed in the future. Combined TRPV1 and TRPM8 antagonists such as CPZ or BCTC both fully suppressed the MR-induced Ca^2+^-influx. This supports the assumption that TRPV1 and TRPM8 are involved in the MR-induced Ca^2+^ increase. Notably, Kwon et al. (2018) determined the immunologic functions of TRPA1 or TRPV1 in allergic conjunctivitis. They concluded that a TRPV1 antagonist rather than a TRPA1 antagonist may ameliorate allergic conjunctivitis by suppressing the Type 2 helper T cell cytokine response in draining lymph nodes [108]. 

### 4.5. Crosstalk between C5a and MR

C5a initiates the production and release of cytokines, mostly in immune cells. IL-6, for instance, can be produced by epithelial cells as well. Anaphylatoxins such as C5a are also known to be part of allergic reactions, especially regarding asthma [109,110,111]. Those can be triggered by the C5a-related release of IL-17 [112], MIP-1α [113], and MIP-2 [114]. New findings showed that C5a has a protective role in fungal infections with *Candida albicans* [115]. In mucormycosis, the complement system, including the anaphylatoxins C5a and C3a, was described as essential to reducing mucor species that have a major impact on clinical outcome [116]. Downregulation of C5a receptors was associated with mucormycosis in trauma patients [117]. As previously described, spores of Mucor (e.g., *Mucor ramosissimus*) seem to activate C5a [118]. In this study, a Ca^2+^-influx was observed after C5a application in the presence of MR (Figure 6a). In contrast, C5a inhibited MR-induced Ca^2+^ increase (Figure 6b). This might present a new aspect of immunoreaction to MR in a conjunctival sample. It shows how the initial immune system can immediately react to pathogens affecting the epithelial layer of the conjunctiva. Therefore, inflammation and complement activation could potentially play a crucial role in defense against pathogens, homeostasis, and wound healing [119]. The novelty is that C5a is able to stimulate TRPV1, whereby TRPV1 activation is known to release pro-inflammatory cytokines in corneal epithelial cells [120], and this may also be the case in HCjEC.

### 4.6. C5a and MR Function on the Cell Membrane

The results of the calcium imaging studies indicate that the C5a-induced Ca^2+^ effects take place at the cell membrane. To investigate calcium regulation specifically at the cell membrane, many of the previous calcium imaging experiments were repeated using the planar patch-clamp technique, which has been established for many years for high-throughput ion channel measurements [121]. First, the effect of C5a on whole-cell currents in HCjEC was measured with and without the aforementioned TRP channel blockers. In brief, the results are congruent with the calcium imaging data and confirm the previous findings (Figure 7, Figure 8, Figure 9, Figures S2 and S3). Both MR and C5a significantly increased the whole-cell currents, confirming the activation of non-selective cation channel currents. The reversal potential fluctuates around 0 mV. The fluctuations are probably due to drift problems (see limitations), but the current response patterns are typical for TRP-like outwardly rectifying currents, even if they are not as clearly visible as in studies with TRPV1- or TRPM8-transfected cells [43]. Studies with human corneal epithelial cells and conjunctival epithelial cells as well as meibomian gland epithelial cells show clear similarities at a similar level [39,60,122,123]. In addition, crosstalk effects were also observed at the cell membrane. For example, MR suppressed the whole-cell currents after they had previously been increased with C5a, which corresponds with a block of the MR-induced Ca^2+^ increase when cells were pre-treated with C5a. This is in line with the calcium imaging experiments (compare Figure 10 with Figure 6c). One possible explanation could be that C5a initially activates TRPs through GPCRs such as C5aR1, which in turn increases intracellular Ca^2+^, as shown in a study in human corneal keratocytes using La^3+^ as a broad TRP Ca^2+^ channel blocker [91]. Subsequently, MR can suppress the C5a effect. Interestingly, a reverse effect can be seen when TRPs are first activated via MR and C5a is added afterwards (Figure S4). The currents then increase significantly once again. This indicates a complex crosstalk between C5a, MR, and the TRPs regarding the activation of the TRPs and the associated calcium regulation (Figure 11). In summary, the sequence of addition of MR and C5a alters the Ca^2+^ response pattern. 

### 4.7. Clinical Relevance

This study can help to better understand the initial immune response of C5a to pathogens such as MR, for example, in allergic or infective conjunctivitis. In further trials, other pathogens or immune components could be interrogated to look for similar reactions. The results might help to develop new medications for conjunctivitis, not only treating symptoms but also acting against the pathogen itself. Those might be C5a-analogues that act to inhibit MR-reaction in epithelial cells, suppressing severe infections in connection with TRPs such as TRPV1 [108]. TRPV1 or TRPM8 antagonists could be considered therapeutics against MR as well. C5a is involved in various ocular pathogeneses [23,124] especially in inflammatory pain associated with the sensitization of TRPV1 [19,125]. To inhibit such responses, C5a could be medically suppressed. Therefore, TRP-antagonists as well as C5L2p can be considered in the future. There are potential clinical benefits, and there are implications that C5L2p (or other proteins) neutralizes C5a [61]. For example, Basta et al., indicate that exogenous immunoglobulin also binds anaphylatoxins C3a and C5a, thereby neutralizing their pro-inflammatory effects [126]. In line with our study, they showed that C5a (as well as C3a) increased intracellular Ca^2+^ in HMC-1 human mast cells. Instead of C5L2p, they used a high-dose of intravenous immunoglobulin (IVIG), which inhibited the C5a-induced rise in intracellular Ca^2+^. In conclusion, Basta et al. suggested that binding of immunoglobulin with C5a (and C3a) may result in neutralization of their biological effects in vitro and in relevant animal models of anaphylatoxin-mediated pathology. These effects may be attributable to a novel effector function of endogenous immunoglobulin and also explain part of the anti-inflammatory effect. Such a possibility may provide therapeutic benefit in a variety of (auto)immune diseases [126]. Similarly, another study by Fung et al. showed that pre-neutralization of C5a with a monoclonal antibody (mAb 137–26) may have therapeutic potential in inflammatory diseases [127]. Therefore, it is conceivable that C5L2p could be directly used as a drug against pathogens, similar to IVIG or monoclonal antibodies, which bind or neutralize C5a and could thus prevent excessive immune reactions. Excessive immune reactions are also triggered via the TRPs, and they lead to prolonged inflammation and hypersensitivity to inflammation. 

Both C5aR1 and C5aR2 (C5L2) receptors are involved in the physio-pathological functions of various ocular diseases, such as retinitis pigmentosa (C5L2) [128], human and experimental cerebral malaria (only C5aR1) [129], or choroidal neovascularization (in mice) [130]. It is noteworthy that TRPV1 can be activated by (mild) hyperosmolarity, resulting in the release of pro-inflammatory cytokines and a reduction in cell shrinkage in human corneal and conjunctival epithelial cells [120,123]. Since C5L2p inhibits the increase in Ca^2+^ concentration mediated by C5a via TRPV1, this approach may be applied in the development of a local (adjuvant) therapy for dry eye disease (DED) or ocular wounds and/or fibrosis [61]. There is a possibility that the use of drugs that inhibit TRPV1 activation may provide a therapeutic benefit to DED patients. This possibility exists because blockage of TRPV1 activation with L-carnitine provides a therapeutic benefit to these individuals [126,131]. A similar approach is suggested in corneal wound healing [132]. However, more studies need to be performed to confirm the use of drugs that block TRPV1 and TRPM8 activation in a clinical setting.

### 4.8. Limitations of This Study

A distinction must first be made between biological and technical limitations. The latter are particularly sensitive, especially regarding the patch-clamp recordings. Leak currents, voltage drifts, and access resistances can change during the measurements. This can be recognized, for example, by the fact that the currents increase significantly after a solution change, even though a blocker has been added. The blocker effect, or trend reversal, was always delayed. In addition, the HCjEC are very fragile cells, so the sealing resistance also changed in between. Very recently, Keller et al. also observed the same phenomenon in meibomian gland epithelial cells, where the patch-clamp measurements also had to be interrupted for a short time [60]. Similarly, many patch-clamp recordings also had to be interrupted in this study so that a new leak subtraction and compensation of the capacitive currents could be carried out in the time gap between the actual measurements. This opportunity was also used to manually improve the contact between cell and chip by temporarily lowering the holding potential (e.g., to −30 mV). In most cases, the seal could then be improved, and the actual recording could be continued. In the time course diagrams, these time gaps at which compensations were carried out are shown as axis breaks (approx. 1 min), in which control measurements were carried out before the actual measurement was continued (e.g., Figure 7, Figure 8 and Figure 9). There were also limitations in the calcium imaging recordings. One of these was a bleaching effect that occurred, for example, when the cells were overloaded with fura-2, or the incubation time was exceeded. However, the bleaching effects could be compensated with the help of a drift correction routine in the TIDA software. A clear biological limitation was that these measurements were performed under in vitro electrophysiological conditions, which do not occur in nature (e.g., ambient temperature lower than body temperature and ambient CO_2_ below physiological CO_2_ level). Therefore, extreme caution should be exercised when trying to extrapolate in vitro data obtained with the IOBA-NHC cell line (HCjEC) to the normal situation, especially in vivo. Nevertheless, the non-transfected, spontaneously immortalized IOBA-NHC cell line is a well-established cell line. Morphologic and functional characterization of the IOBA-NHC cell line shows that this cell line may be a useful experimental tool in the field of ocular surface cell biology [63]. In addition to these limitations, there are also strengths to this study. On the one hand, the extremely high repetition rates with high n-numbers that sometimes reach into the three-digit range for the calcium imaging recordings should be mentioned here. It should also be mentioned that some of the measurements presented in this study were repeated by independent students during their lab internships. The extremely high data replication (also for the patch-clamp recordings) strengthens the reproducibility of the data and thus the conclusions.

### 4.9. Conclusions

Functional assays such as fluorescence calcium imaging and the planar patch-clamp technique were successfully applied in this study to elucidate complex interactions between C5a, MR, and TRPV1 or TRPM8 in modulating rises in Ca^2+^ influx. Our findings indicate an association between the anaphylatoxin C5a and TRPs such as TRPV1 and TRPM8 for the first time in HCjEC. Moreover, the most striking finding was that the N-terminal peptide C5L2p was able to inhibit the C5a receptor-mediated response to C5a by neutralizing C5a. This led to a decrease in C5a-induced Ca^2+^ influx and whole-cell currents. We also identified crosstalk between C5a and MR. This interaction controls human conjunctival cell function through modulating interactions of TRPV1 with TRPM8 channel activity. Overall, the implications of the findings are that C5L2p neutralizing C5a activation may provide a potential clinical benefit in offsetting inflammation. This could represent a novel effector function of C5L2p and also explain part of its anti-inflammatory effect in a variety of autoimmune diseases.

## Data Availability

The data presented in this study are available on request from the corresponding author. The data is not publicly available due to privacy limitations.

## Supplementary Materials

The following supporting information can be downloaded at: https://www.mdpi.com/article/10.3390/cells13161329/s1, Figure S1: MR-induced Ca^2+^-increase abolished in Ca^2+^ free RLS; Figure S2: C5a increased whole-cell currents through TRPM8; Figure S3: MR increased whole-cell currents through TRPV1. Figure S4: MR amplify C5a-induced increase of whole-cell currents; Figure S5: C5a induced influx in the presence of external Ca^2+^; Figure S6: HCjEC microscopic images.
