# Supplementary material for: Complement Component C5a and Fungal Pathogen Induce Diverse Responses through Crosstalk between Transient Receptor Potential Channel (TRPs) Subtypes in Human Conjunctival Epithelial Cells
Source: Cells. 2024 Aug 9;13(16):1329. doi: 10.3390/cells13161329 (PMC11352353; doi:10.3390/cells13161329)
Supplement: Supplementary file 1 [file cells-13-01329-s001.zip › cells-3105678-supplementary.pdf]

Supplementary Materials:

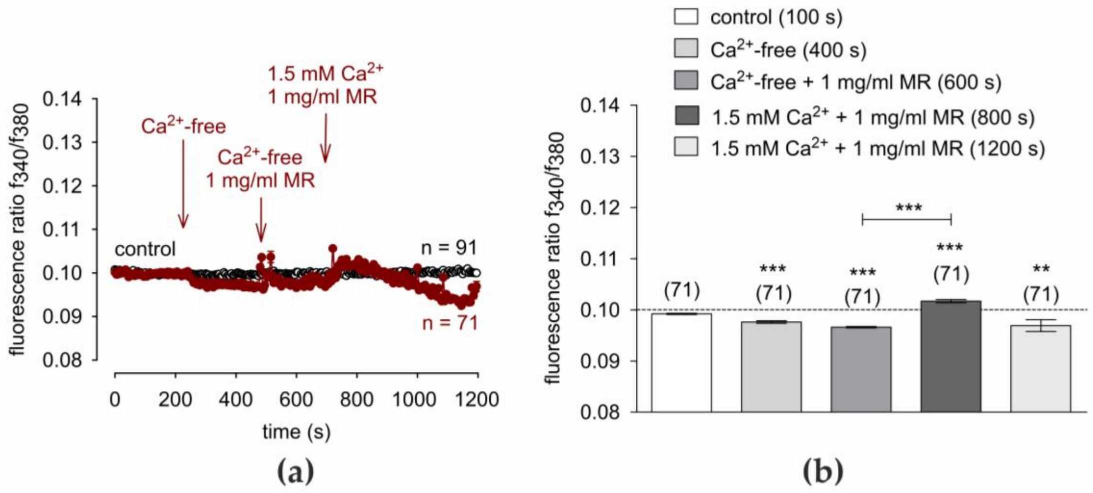

Figure S1. MR-induced Ca^{2+}-increase abolished in Ca^{2+} free RLS. Data are means ± SEM. The reagents were added at the time points indicated by arrows. **(a)** Application of 1 mg/ml MR had no clear effect on Ca^{2+} regulation. It fluctuates around the baseline ($n = 71$, red filled circles). In presence of extracellular Ca^{2+}, non-treated control cells maintained a constant Ca^{2+}-baseline ($n = 91$, open circles). **(b)** Summary of the experiments with MR in HCjEC with and without extracellular Ca^{2+}. The dashed line is the reference line at 0.10. The asterisks (**) and (***) designate significant decrease in $[Ca^{2+}]_i$ in lack of extracellular Ca^{2+} with and without MR ($t = 400$ s, 600 s; $n = 71$; **$p < 0.01$; ***$p < 0.001$; paired tested) compared to control ($t = 100$ s) as well as an increase in $[Ca^{2+}]_i$ in presence of extracellular Ca^{2+} after MR application ($t = 800$ s; $n = 71$; ***$p < 0.001$; paired tested) and a following decrease in $[Ca^{2+}]_i$ ($t = 1200$ s; $n = 71$; **$p < 0.01$; paired tested) compared to control ($t = 100$ s).

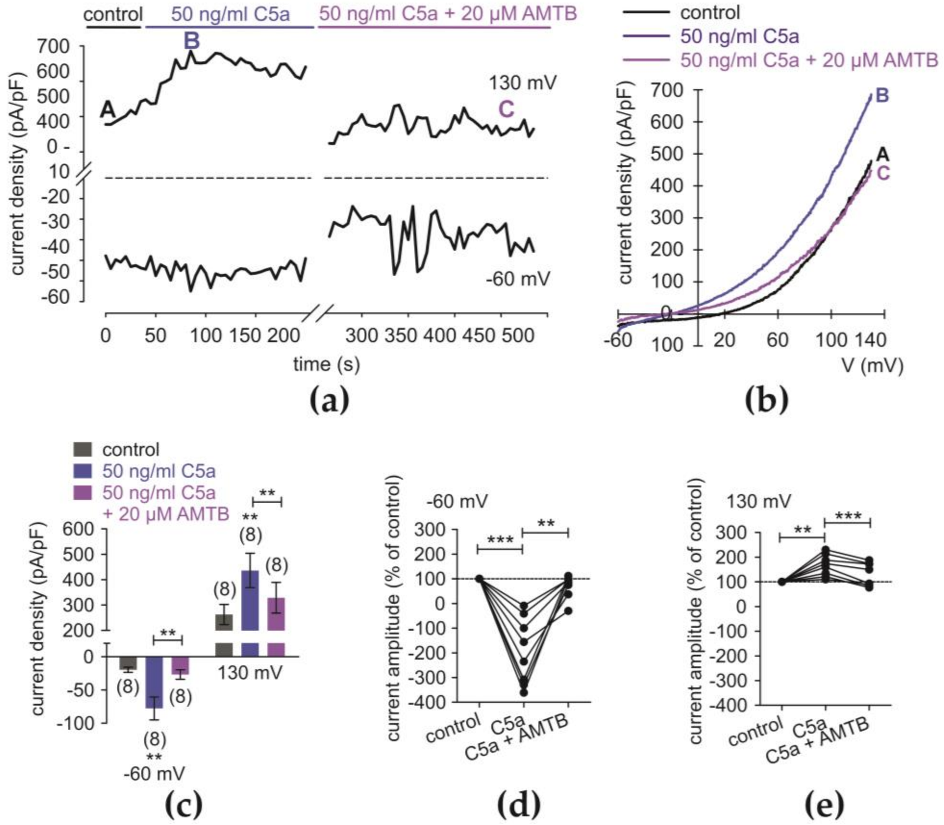

Figure S2. C5a increased whole-cell currents through TRPM8. **(a)** Time course recording of the current increases induced by C5a (50 ng/ml) and declined after application of AMTB (20 µM). The dashed line is the reference line at 0 pA/pF. **(b)** Original traces of C5a-induced current responses to voltage ramps. Current densities are shown before application as control (labelled as A), during application of 50 ng/ml C5a (labelled as B) and after addition of 20 µM AMTB (labelled as C). **(c)** Summary of the experiments with C5a and AMTB. The asterisks (**) indicate statistically significant differences of whole-cell currents with and without C5a ($n = 8$; ** $p < 0.01$; paired tested) and significant differences of C5a-induced rises with and without AMTB ($n = 8$; ** $p < 0.01$; paired tested). **(d)** Maximum negative current amplitudes induced by a voltage step from 0 to −60 mV are expressed in percent of control values before application of 50 ng/ml C5a (control set to 100%; dashed line). C5a-induced inward currents could be clearly suppressed in the presence of 20 µM AMTB. The asterisks (**) and (***) indicate statistically significant differences of whole-cell currents with and without C5a ($n = 8$; **$p < 0.01$, ***$p < 0.001$; paired tested) and significant differences of C5a-induced rises with and without AMTB ($n = 8$; ** $p < 0.01$, ***$p < 0.01$; paired tested). **(e)** Same diagram as shown in (d) but related to maximum positive current amplitudes induced by a voltage step from 0 to +130 mV. 20 µM AMTB has an inhibitory effect on C5a-induced outward currents.

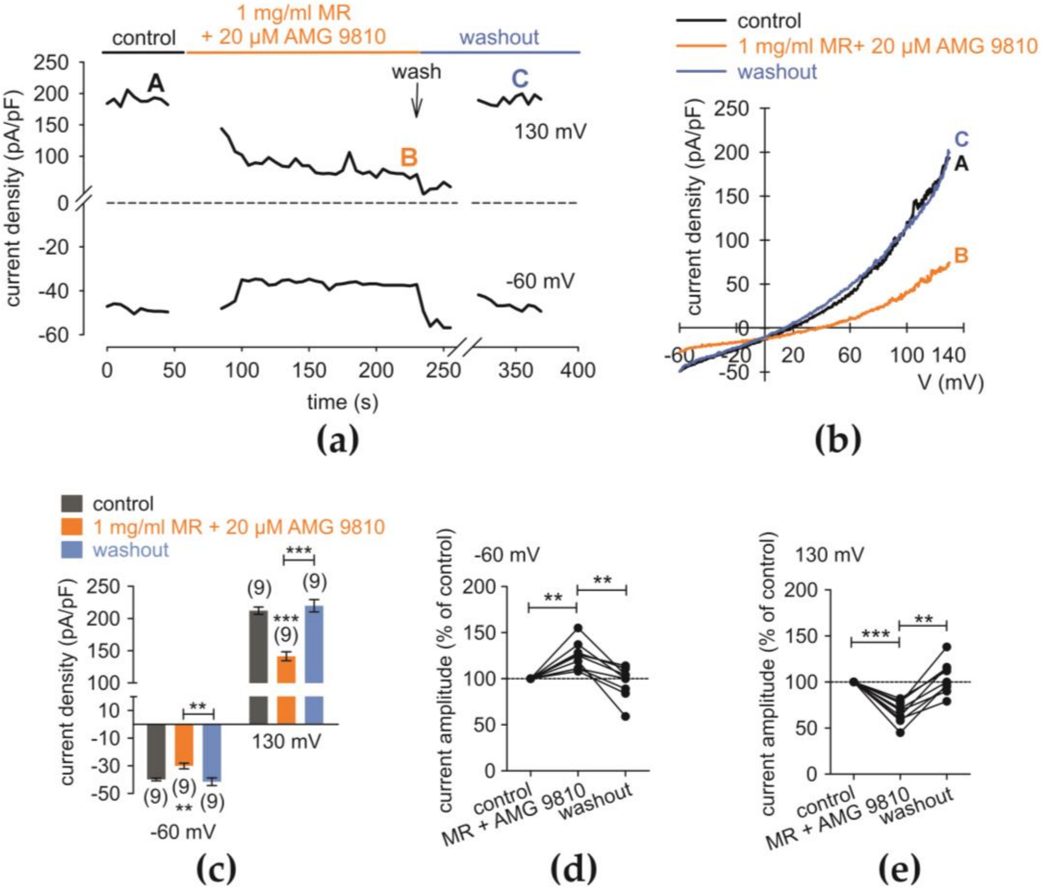

Figure S3. MR increased whole-cell currents through TRPV1. **(a)** Time course recording of the current increases induced by MR (1 mg/ml) and declined after application of AMG 9810 (20 µM). The dashed line is the reference line at 0 pA/pF. **(b)** Original traces of MR-induced current responses to voltage ramps. Current densities are shown before application as control (labelled as A), during application of 1 mg/ml MR (labelled as B) and after addition of 20 µM AMG 9810 (labelled as C). **(c)** Summary of planar patch-clamp experiments with MR + AMG 9810. The asterisks (**) and (***) indicate statistically significant differences of whole-cell currents with and without MR + AMG 9810 ($n = 9$; **$p < 0.01$, ***$p < 0.001$; paired tested) **(d)** Maximum negative current amplitudes induced by a voltage step from 0 to −60 mV are expressed in percent of control values before application of 1 mg/ml MR + 20 µM AMG 9810 (control set to 100%; dashed line). The asterisks (**) indicate statistically significant differences of whole-cell currents with and without MR + AMG 9810 ($n = 9$; **$p < 0.01$; paired tested). MR + 20 µM AMG 9810 reduced inward currents in comparison to control and washout. **(e)** Same diagram as shown in (d) but related to maximum positive current amplitudes induced by a voltage step from 0 to +130 mV.

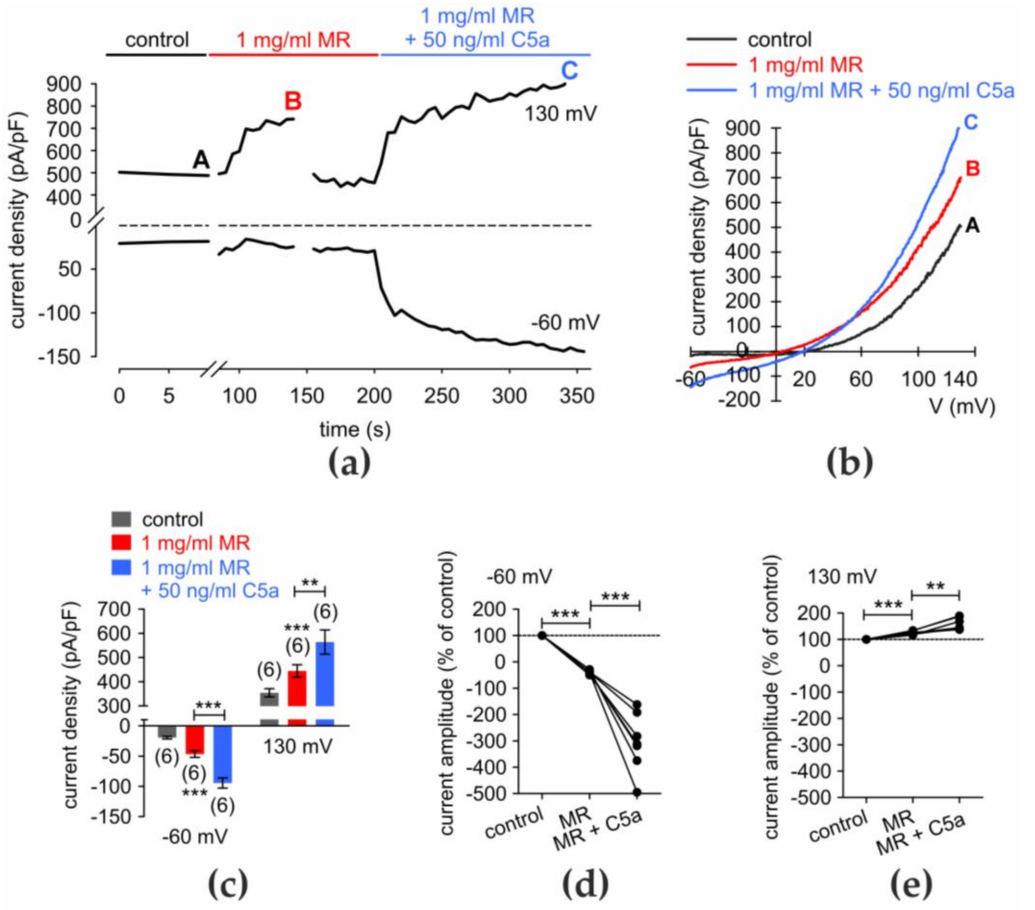

Figure S4. MR amplify C5a-induced increase of whole-cell currents. **(a)** Time course recording of the current increases induced by MR (1 mg/ml) and additional C5a (50 ng/ml). The dashed line is the reference line at 0 pA/pF. **(b)** Original traces of MR and C5a-induced current responses to voltage ramps. Current densities are shown before application as control (labelled as A), during application of 1 mg/ml MR (labelled as B) and after addition of 50 ng/ml C5a (labelled as C). **(c)** Summary of the experiments with MR + C5a. The asterisks (**) and (***) indicate statistically significant differences of whole-cell currents with and without MR and added C5a ($n = 6$; ** $p < 0.01$, *** $p < 0.001$; paired tested) **(d)** Maximum negative current amplitudes induced by a voltage step from 0 to −60 mV are expressed in percent of control values before application of 1 mg/ml MR (control set to 100%; dashed line). MR increased inward currents in comparison to control while C5a-induced increase is amplified by MR. The asterisks (***) indicate statistically significant differences of whole-cell currents ($n = 6$; *** $p < 0.001$; paired tested) **(e)** Same diagram as shown in (d) but related to maximum positive current amplitudes induced by a voltage step from 0 to +130 mV.

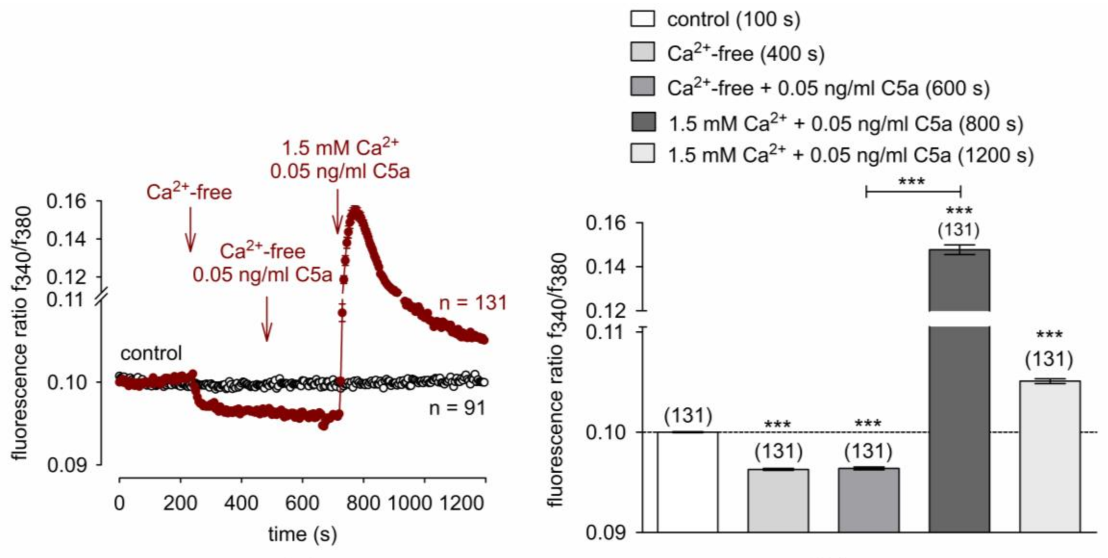

(a) (b)

Figure S5. C5a induced influx in the presence of external Ca^{2+}. Data are means ± SEM. The reagents were added at the time points indicated by arrows. **(a)** In absence of extracellular Ca^{2+}, the Ca^{2+} decreased below the baseline level after application of C5a (0.05 ng/ml). However, there was a considerably high Ca^{2+} transient when external Ca^{2+} was added (RLS) ($n = 131$, red filled circles). Non-treated control cells maintained a constant Ca^{2+}-baseline ($n = 91$, open circles). **(b)** Summary of the experiments with C5a in HCjEC with and without extracellular Ca^{2+}. The asterisks (***) designate significant decrease in $[Ca^{2+}]_i$ in lack of extracellular Ca^{2+} with and without C5a ($t = 400$ s, 600 s; $n = 131$; ***$p < 0.001$; paired tested) compared to control ($t = 100$ s) as well as an increase in $[Ca^{2+}]_i$ in presence of extracellular Ca^{2+} after C5a application ($t = 800$ s, 1200 s; $n = 131$; $p < 0.001$; paired tested) compared to control ($t = 100$ s).

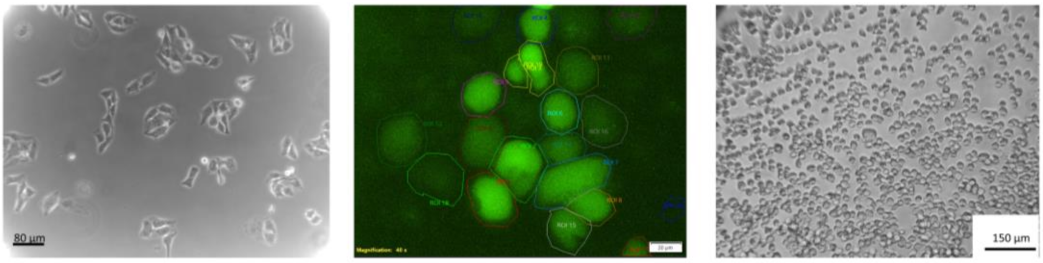

(a) (b) (c)

Figure S6. HCjEC microscopic images **(a)** Light microscopic image of HCjEC (IOBA-NHC) cells. Scale bar is 80 µm. **(b)** Fura-2 loaded HCjEC (IOBA-NHC) cells visible using fluorescence emission at 510 nm (green fluorescence light) shortly before the experiment. Cells were used in this configuration to perform calcium imaging. Scale bar is 20 µm. **(c)** Light microscopic image of HCjEC (IOBA-NHC) cells in suspension as used for planar patch-clamp recordings. Scale bar is 150 µm.
